# Norovirus in Bottled Water Associated with Gastroenteritis Outbreak, Spain, 2016

**DOI:** 10.3201/eid2309.161489

**Published:** 2017-09

**Authors:** Albert Blanco, Susana Guix, Noemí Fuster, Cristina Fuentes, Rosa Bartolomé, Thais Cornejo, Rosa Maria Pintó, Albert Bosch

**Affiliations:** University of Barcelona, Barcelona, Spain (A. Blanco, S. Guix, N. Fuster, C. Fuentes, R.M. Pintó, A. Bosch);; Hospital Universitari Vall d'Hebron, Barcelona (R. Bartolomé, T. Cornejo)

**Keywords:** norovirus, bottled water, outbreak, gastroenteritis, viruses, Spain, Catalonia, enteric infections

## Abstract

In April 2016, an outbreak of gastrointestinal illness (4,136 cases) occurred in Catalonia, Spain. We detected high levels of norovirus genotypes I and II in office water coolers associated with the outbreak. Infectious viral titer estimates were 33–49 genome copies/L for genotype I and 327–660 genome copies/L for genotype II.

During April 11–25, 2016, a total of 4,136 cases of gastroenteritis were reported by the Public Health Agency of Catalonia (ASPCAT; Figure, panel A). A case-patient was defined as an exposed person who had vomiting or diarrhea (3 or more loose stools within 24 hours) and >2 of the following: nausea, abdominal pain, or fever (≥37.8°C). Six patients required hospitalization. 

**Figure:**
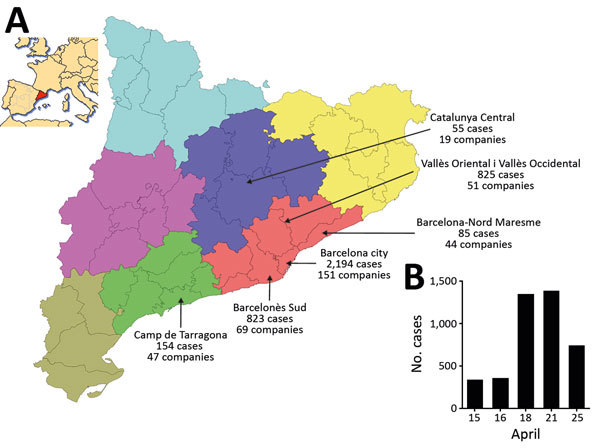
Waterborne norovirus outbreak in Catalonia, Spain, April 15–25, 2016 (n = 4,136 cases). A) Geographic distribution of the number of cases and affected companies in the Catalonian Health regions. Inset shows location of region in Spain. Map outlines obtained from https://commons.wikimedia.org/wiki/File:Catalonia_location_map.svg. B) Time distribution of reported cases. Cases are displayed according to the dates of the press release from the Public Health Agency of Catalonia (http://premsa.gencat.cat/pres_fsvp/AppJava/notapremsavw/292423/ca/salut-publica-dona-tancat-brot-gastroenteritis-transmes-consum-daigua-envasada.do). Although the onset of the outbreak was on April 11, the first report of the number of cases was on April 15, and the outbreak was declared over on April 25 with a total of 4,136 reported cases, including both primary and secondary cases.

The epidemiologic investigation conducted by the ASPCAT pointed toward an association of the outbreak with drinking bottled spring water from office water coolers; the water had been bottled at a source in Andorra (M. Jané-Checa and A. Martínez-Mateo, Public Health Agency of Catalonia, pers. comm., 2016 Sep 1). Compared with other modes of transmission such as food or person to person, norovirus outbreaks associated with drinking water are rare in developed countries (*1*). On April 15, 2016, as a precautionary measure, the company producing the bottled water recalled >6,150 containers of water of suspected quality that had already been distributed to 925 companies. The water complied with all requirements of the European Commission directive on the exploitation and marketing of natural mineral waters (*2*), but these requirements do not include any virologic determination.

The Spanish Authority for Consumption, Food Safety, and Nutrition reported the outbreak at the national (http://www.aecosan.msssi.gob.es/AECOSAN/web/seguridad_alimentaria/ampliacion/gastroenteritis_agua_envasada.htm) and European (Rapid Alert System for Food and Feed, RASFF, expedient 2017/0469, https://webgate.ec.europa.eu/rasff-window/portal/?event = notificationDetail&NOTIF_REFERENCE = 2016.0469) levels. The numbers of cases reported by the ASPCAT peaked on April 18 and 21 (Figure, panel B), and the ASPCAT declared the outbreak over on April 25.

## The Study

As part of the epidemiologic investigation of this outbreak, we took samples from four 19-L water coolers in 2 offices in the Barcelona metropolitan area, from which affected persons had drunk. We collected samples 1 and 2 on April 15 from 2 water coolers in 1 office, from which 36 cases had been reported. A private company provided samples 3 and 4, from 2 water coolers in a different office with an unknown number of cases, on April 20. We tested all samples immediately upon receipt at our laboratory. We used positively charged glass wool and polyethylene glycol precipitation for virus concentration. Sample volumes ranged from 2.0 L to 7.8 L; we reduced each sample to a final volume of 7 mL, as described previously (*3*). We extracted total RNA from 0.5 mL of the concentrates with the NucliSens miniMAG magnetic system (BioMérieux, Marcy-l’Étoile, France) and eluted the samples in 100 µL of elution buffer, following the manufacturer’s specifications. We performed a standardized 1-step real-time TaqMan reverse transcription PCR (RT-qPCR; Ultrasense, Invitrogen Life Technologies, Barcelona, Spain), in which we used 5 µL of extracted RNA to determine the number of genome copies per liter of human norovirus genogroup I (GI) and genogroup II (GII) (*4**–**7*). We monitored virus/nucleic acid extraction and enzyme efficiencies as previously described; we used double-stranded DNA plasmids containing the target sequences as standards (*8*).

We detected high RNA levels for norovirus GI and GII, around 10^3^ and 10^4^ genome copies/L, in 2 of the 4 water cooler samples concentrated by glass wool filtration and polyethylene glycol precipitation (Table). Because molecular methods are unable to discern between infectious and noninfectious particles, we predicted the infectivity of norovirus in the concentrated samples by treating the samples with the nucleic acid intercalating dye PMA propidium monoazide; (50 µmol/L) and Triton X surfactant (0.5%) before RT-qPCR; this enabled us to distinguish between virions with intact and altered capsids (*9*). Following this approach, estimated infectious levels in the 2 positive samples were 49 and 327 genome copies/L for norovirus GI and 33 and 660 genome copies/L for norovirus GII (Table).

**Table:** Human norovirus genome copies per liter in analyzed water cooler samples concentrated by glass wool filtration and polyethylene glycol precipitation, metropolitan area of Barcelona, Spain, April 15–20, 2016*

Sample	RT-qPCR			PMA/T	
	GI	GII		GI	GII
1	1.1 × 10^3^	5.8 × 10^3^		49	327
2	1.0 × 10^4^	2.6 × 10^4^		33	660
3	ND	ND		NT	NT
4	ND	ND		NT	NT

*G, genogroup; ND, not detected; NT, not tested; PMA/T, treatment with the nucleic acid intercalating dye propidium monoazide (PMA, 50 µmol/L) and Triton X surfactant (0.5%) before RT-qPCR; RT-qPCR, real-time TaqMan reverse transcription PCR.

Given the large number of persons involved in the outbreak and the reported 50% human infectious dose for norovirus of 18–1,300 particles (*10**,**11*), the high genome copy values in the water samples were not unexpected. In addition, the proportion of intact (infectious) virions in the water samples, estimated through PMA/Triton treatment before RT-qPCR assays, represented 0.3%–5.6% of the total number of physical particles, which was enough to cause gastrointestinal illness (*10**,**11*).

We assayed the presence of enteroviruses, astroviruses, sapoviruses, rotaviruses, adenoviruses, and hepatitis A virus in the 4 water samples by using commercial RT-qPCR kits (Viasure, Certest Biotec SL, Zaragoza, Spain), with negative results. We attempted genotyping of noroviruses in samples 1 and 2 using a semi-nested RT-PCR protocol with specific primers for GI and GII. We performed the first PCR with primers COG1F and G1SKR for GI and COG2F and G2SKR for GII (*6**,**12*). For the second PCR, we used primers G1SKF and G1SKR for GI and G2SKF and G2SKR for GII (*12*). We assigned genotypes based on clustering with reference strains from the sequence database of the European network NoroNet and norovirus genotyping tool (*13*). We detected a single sequence corresponding to genotype GII.4/Sydney/2012 (GenBank accession no. KX816644) in samples 1 and 2. Additionally, MiSeq next-generation sequence analysis (Illumina, San Diego, CA) of the amplified product confirmed the sole presence of genotype GII.4/Sydney/2012 (data not shown). 

Although some fecal samples from persons who worked at the office from which water samples 1 and 2 were obtained contained genotypes GI.2 (n = 10) and GII.17 (n = 11) but not GII.4/Sydney/2012, we isolated genotypes GII.4/Sydney/2012 (n = 1), GI.2 (n = 1), GII.17 (n = 1), and GII.2 (n = 1) from fecal samples from persons from a different office who exhibited the same gastrointestinal symptoms after drinking water supplied by the same company (data not shown). We hypothesize that the spring water was contaminated by all 4 strains (GI.2, GII.2, GII.4, and GII.17) but levels of viral contamination for each genotype were not homogeneous in all bottled coolers. We may have detected only the GII.4 genotype in water samples 1 and 2 because of a higher concentration of this specific genotype or because of bias caused by the sampling, concentration, and molecular detection procedures. Finally, several reasons could explain why we did not find any GII.4/Sydney/2012 or GII.2 genotypes among the fecal samples from persons from the office that provided samples 1 and 2, including the existence of immune status among the exposed persons or differences in the proportion of infectious/physical particles between the different types.

## Conclusions

We describe quantitative detection of norovirus in bottled water. Previously, several brands of mineral water were reported to contain norovirus, but the findings were later attributed to laboratory contamination with control reagents (*14**,**15*). One limitation of our study is the low number of water samples analyzed. Four days after the onset of the outbreak, the company recalled all batches of water and water coolers of suspected quality, which hampered the collection of a larger number of samples for analysis.

The cause of the water contamination remains to be elucidated. However, the high number of affected persons from 381 offices that received water coolers, and the many different genotypes found in some patients’ fecal specimens, point toward sewage pollution of the spring aquifer. Aquifer pollution was acknowledged by the Andorra Ministry of Health and Welfare, and further use of the spring was banned.

This large outbreak suggests that the management of microbial risks of commercially produced mineral waters, universally based solely on bacterial parameters, could benefit from additional analysis for relevant viral pathogens such as norovirus. However, the substantial costs incurred in developing, enhancing, and managing virus surveillance systems call for a balanced approach to keep both the cost and the time required for the analyses within feasibility limits.
