# Correction: Vol. 23, No. 9

**DOI:** 10.3201/eid2311.C12311

**Published:** 2017-11

**Keywords:** errata, erratum, correction

Norovirus genogroups were referred to incorrectly in the abstract of Norovirus in Bottled Water Associated with Gastroenteritis Outbreak, Spain, 2016 (A. Blanco et al.). The article has been corrected online (https://wwwnc.cdc.gov/eid/article/23/9/16-1489_article).

